# Editorial: Clinical utilization of plant-based nutrition and fasting protocols as novel therapies

**DOI:** 10.3389/fnut.2024.1390857

**Published:** 2024-03-13

**Authors:** Humaira Jamshed, Nick Wright, Owen Kelly

**Affiliations:** ^1^Integrated Science and Mathematics, Dhanani School of Science and Engineering, Habib University, Karachi, Pakistan; ^2^Royal New Zealand College of General Practitioners, Wellington, New Zealand; ^3^Department of Molecular and Cellular Biology, College of Osteopathic Medicine, Sam Houston State University, Conroe, TX, United States

**Keywords:** plant-based nutrition, clinical applications, dietary quality and intake patterns, therapeutic interventions, fasting protocols

## Introduction

In contemporary healthcare the exploration of innovative therapies that integrate ancient wisdom with modern scientific inquiry is essential. Notably, plant-based nutrition and fasting protocols have gathered attention as emerging approaches reviving ancestral practices and offering a promising frontier in therapeutic innovation. This Research Topic aims to bring together the insights from five pivotal articles, elucidating the interconnectedness of these studies and their collective contribution to the expanding field of diet-based interventions in clinical practice, in both the hospital and outpatient settings.

### Fasting-mimicking diet

The bibliometric analysis of FMDs serves as the foundation, providing an overarching view of the research landscape (Lin and Gao). This study highlights the growing interest in FMDs and identifies the key areas such as cancer, metabolic diseases, and cognitive improvement as hotspots of current research. The key contributing factor in these diets may be their plant-based nature and their focus on nutrient density. While the biological mechanisms supporting FMDs are not fully elucidated, the emphasis on FMD's potential benefits sets a foundational context for understanding the subsequent exploration of plant-based diets.

### Plant-based diet indices and metabolic syndrome

Building on the groundwork laid by the FMD analysis, the systematic review of plant-based diet indices and MetS explores specific dietary patterns within the broader spectrum of plant-based nutrition (Nikparast et al.). By differentiating between healthy and unhealthy plant-based diets, this study emphasizes the importance of diet quality and composition, echoing the selective nourishment principle of FMD's strategic calorie restriction. Diets that can address MetS have a much broader potential to address inflammation, insulin resistance, and other metabolic dysfunctions associated with chronic disease.

### Impact on sleep

The mini-review on plant-rich diets and sleep introduces another dimension to the discussion, linking dietary patterns with sleep quality (Polianovskaia et al.). This connection is critical, as both FMDs and healthy plant-based diets have been associated with systemic health improvements, which in turn, could influence sleep patterns and overall wellbeing. The article bridges dietary interventions with sleep, an often-overlooked aspect of health. Obtaining good sleep has been recently added to the American Heart Association's Life's Simple 7™ (to become Life's Essential 8™) highlighting the importance of sleep in overall health. The connection between nutrition and sleep quality adds a new dimension to our understanding, suggesting that what we eat not only fuels our body but also nurtures our sleep, and plant-based nutrition may help achieve this.

### Anthocyanin supplementation

Anthocyanins are water-soluble red/blue pigments present in whole fruits and vegetables, with antioxidant and antimicrobial properties, and may modulate the intestinal microbiota. The systematic review on anthocyanin supplementation presents a focused exploration of specific plant-derived bioactives and their impact on blood lipid levels (Jang et al.). This study complements the broader discussions on plant-based diets by providing evidence of the cardiovascular benefits of specific plant components. However, it is plausible to suggest that many other plant compounds contribute to the reduction of CVD risk. This article reinforces the potential of whole-food, plant-based dietary interventions, including FMD in reducing the burden of chronic disease.

### Botanical lozenges for chronic pharyngitis

Finally, the randomized controlled trial investigating botanical lozenges in alleviating throat discomfort addresses the application of plant extracts in treating specific clinical conditions (Wu et al.). This study exemplifies the practical applications of plant-based nutrition and shows the possibilities beyond dietary interventions, extending the therapeutic potential from prevention and systemic health improvement to targeted treatment interventions.

## The emergent picture

The integration of these articles reveals a cohesive narrative: the exploration of plant-based nutrition and fasting protocols not only demonstrates opportunities for chronic disease management and prevention but also highlights the importance of dietary quality and specific bioactive components in achieving therapeutic outcomes. The progression from broad dietary strategies (FMDs and plant-based diets) to specific interventions (anthocyanin supplementation and botanical lozenges) illustrates a multi-layered approach to nutrition-based therapies.

## Challenges and future directions

While the interconnected findings of these articles offer an optimistic view of the future of diet-based interventions, they also signify the need for further research. It is highly probable that diets for clinical conditions would not be similar to standard dietary guidelines for healthy people (e.g., Dietary Guidelines for Americans), and the dependence on these standard guidelines, and the Dietary Reference Intakes, as the default targets for people with health conditions may not be therapeutically beneficial. Furthermore, considering whole foods are composed of thousands of compounds, focusing only on the 30–40 essential nutrients, ignores a world of possible future therapeutic breakthroughs. Key challenges include the need for comprehensive clinical trials to validate the efficacy of these, and other whole-food plant-based interventions, across diverse populations and conditions. In parallel, the investigation of the biochemical mechanisms underpinning the health benefits of fasting and plant-based diets could also be explored. Exploring patient adherence and perception toward diet-based therapies in diverse clinical settings would also be clinically useful.

## Conclusion

The synthesis of insights from these five articles highlights the potential for interdisciplinary research to unravel the complex mechanisms through which diet, specifically whole-food plant-based diets, affect health. These articles collectively underscore the potential of plant-based nutrition and fasting protocols as novel therapeutic strategies. By highlighting the interconnectedness of dietary interventions, from systemic health benefits to targeted treatments, this Research Topic advocates for a nuanced understanding of nutrition's role in healthcare. As the field evolves, further research and clinical application will be crucial in realizing the full potential of these innovative therapies.

## Author contributions

HJ: Writing – original draft, Writing – review & editing. NW: Writing – review & editing. OK: Writing – review & editing.

